# Type 2 diabetes does not account for ethnic differences in exercise capacity or skeletal muscle function in older adults

**DOI:** 10.1007/s00125-019-05055-w

**Published:** 2019-12-09

**Authors:** Siana Jones, Therese Tillin, Suzanne Williams, Sophie V. Eastwood, Alun D. Hughes, Nishi Chaturvedi

**Affiliations:** MRC Unit for Lifelong Health and Ageing at UCL, Department of Population Science and Experimental Medicine, Institute of Cardiovascular Science, University College London, Gower Street, London, WC1E 6BT UK

**Keywords:** Ethnicity, Exercise capacity, Skeletal muscle, Type 2 diabetes

## Abstract

**Aims/hypothesis:**

The aim of this study was to compare exercise capacity, strength and skeletal muscle perfusion during exercise, and oxidative capacity between South Asians, African Caribbeans and Europeans, and determine what effect ethnic differences in the prevalence of type 2 diabetes has on these functional outcomes.

**Methods:**

In total, 708 participants (aged [mean±SD] 73 ± 7 years, 56% male) were recruited from the Southall and Brent Revisited (SABRE) study, a UK population-based cohort comprised of Europeans (*n* = 311) and South Asian (*n* = 232) and African Caribbean (*n* = 165) migrants. Measurements of exercise capacity using a 6 min stepper test (6MST), including measurement of oxygen consumption ($\dot{V}{\mathrm{O}}_2$) and grip strength, were performed. Skeletal muscle was assessed using near infrared spectroscopy (NIRS); measures included changes in tissue saturation index (∆TSI%) with exercise and oxidative capacity (muscle oxygen consumption recovery, represented by a time constant [τ]). Analysis was by multiple linear regression.

**Results:**

When adjusted for age and sex, in South Asians and African Caribbeans, exercise capacity was reduced compared with Europeans ($\dot{V}{\mathrm{O}}_2$ [ml min^−1^ kg^−1^]: β = −1.2 [95% CI –1.9, −0.4], *p* = 0.002, and β −1.7 [95% CI –2.5, −0.8], *p* < 0.001, respectively). South Asians had lower and African Caribbeans had higher strength compared with Europeans (strength [kPa]: β = −9 [95% CI –12, −6), *p* < 0.001, and β = 6 [95% CI 3, 9], *p* < 0.001, respectively). South Asians had greater decreases in TSI% and longer τ compared with Europeans (∆TSI% [%]: β = −0.9 [95% CI –1.7, −0.1), *p* = 0.024; τ [s]: β = 11 [95% CI 3, 18], *p* = 0.006). Ethnic differences in $\dot{V}{\mathrm{O}}_2$ and grip strength remained despite adjustment for type 2 diabetes or HbA_1c_ (and fat-free mass for grip strength). However, the differences between Europeans and South Asians were no longer statistically significant after adjustment for other possible mediators or confounders (including physical activity, waist-to-hip ratio, cardiovascular disease or hypertension, smoking, haemoglobin levels or β-blocker use). The difference in ∆TSI% between Europeans and South Asians was marginally attenuated after adjustment for type 2 diabetes or HbA_1c_ and was also no longer statistically significant after adjusting for other confounders; however, τ remained significantly longer in South Asians vs Europeans despite adjustment for all confounders.

**Conclusions/interpretation:**

Reduced exercise capacity in South Asians and African Caribbeans is unexplained by higher rates of type 2 diabetes. Poorer exercise tolerance in these populations, and impaired muscle function and perfusion in South Asians, may contribute to the higher morbidity burden of UK ethnic minority groups in older age.

**Electronic supplementary material:**

The online version of this article (10.1007/s00125-019-05055-w) contains peer-reviewed but unedited supplementary material, which is available to authorised users.

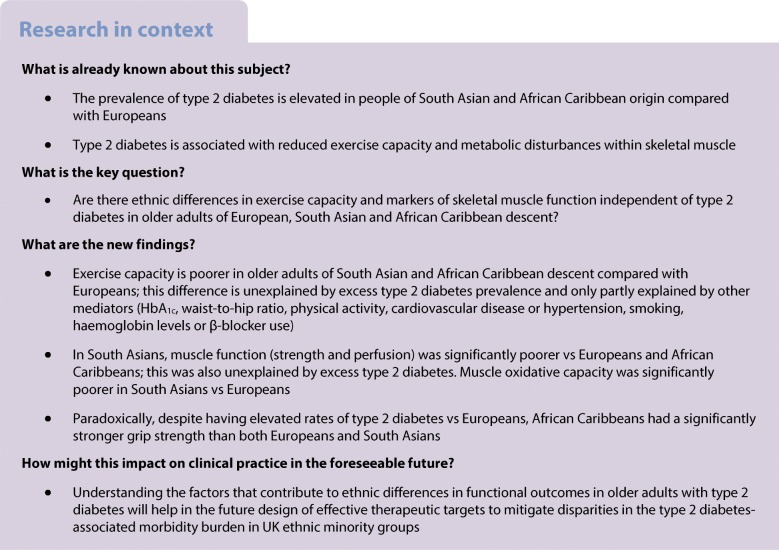



## Introduction

The prevalence of type 2 diabetes is higher in men and women of South Asian and African Caribbean origin compared with Europeans [1], yet the pathophysiological mechanisms underlying these disparities are not fully understood [1, 2]. Given that skeletal muscle is the main site of glucose disposal, abnormal skeletal muscle metabolism is a plausible candidate to explain ethnic differences in susceptibility to type 2 diabetes, and some evidence supports this hypothesis [3–5]. However, there is also evidence to show that type 2 diabetes adversely influences skeletal muscle function, so a bidirectional relationship may exist [6].

At the whole-body level, reduced cardiorespiratory fitness and muscle strength are associated with higher rates of incident type 2 diabetes [7]. Compared with Europeans, South Asians have reduced peak oxygen consumption ($\dot{V}{\mathrm{O}}_{2\mathrm{peak}}$) during exercise and reduced grip strength [4, 5, 8]. Comparisons of skeletal muscle oxidative capacity in skeletal muscle biopsy samples have provided conflicting results, reporting enhanced, similar or diminished levels in South Asians compared with Europeans [8–10]. Studies conducted in the USA report impaired cardiorespiratory fitness [11] and skeletal muscle oxidative capacity [12] in African Americans compared with white people from the USA. However, grip strength does not appear to differ between black African people and individuals of European descent in the UK [5]. Whether the excess risk of type 2 diabetes and, more broadly, hyperglycaemia and obesity, can account for ethnic differences in fitness, muscle function and quality has not been studied.

Local skeletal muscle measurements have generally been carried out through analysis of muscle biopsies to provide in-depth metabolic markers of function. Oxidative capacity is typically described by mitochondrial number, content and function (enzyme activity, gene expression or the ratio of mitochondrial/nuclear DNA). In vivo, oxidative capacity measurements are possible via application of ^31^P-magnetic resonance spectroscopy (^31^P-MRS) or near infrared spectroscopy (NIRS) and, although it is not possible to interrogate oxidative capacity with the same molecular detail as a tissue sample permits, a major advantage of an in vivo study is that its findings better reflect whole-body, interrelated pathways, including important systemic contributions, such as central control. Ethnic differences in exercise capacity and their association with skeletal muscle quality, measured in vivo, have not previously been compared.

Therefore, the objectives of this study were, first, to compare whole-body exercise capacity and skeletal muscle power (grip strength) and in vivo measures of skeletal muscle quality between Europeans, South Asians and African Caribbeans. Second, we wished to explore the role of type 2 diabetes and other potential mediators in accounting for any ethnic differences observed. Finally, we compared correlations between non-invasive muscle measurements and $\dot{V}{\mathrm{O}}_2$ during submaximal exercise between different ethnicity.

## Methods

### Participants

**Fig. 1:**
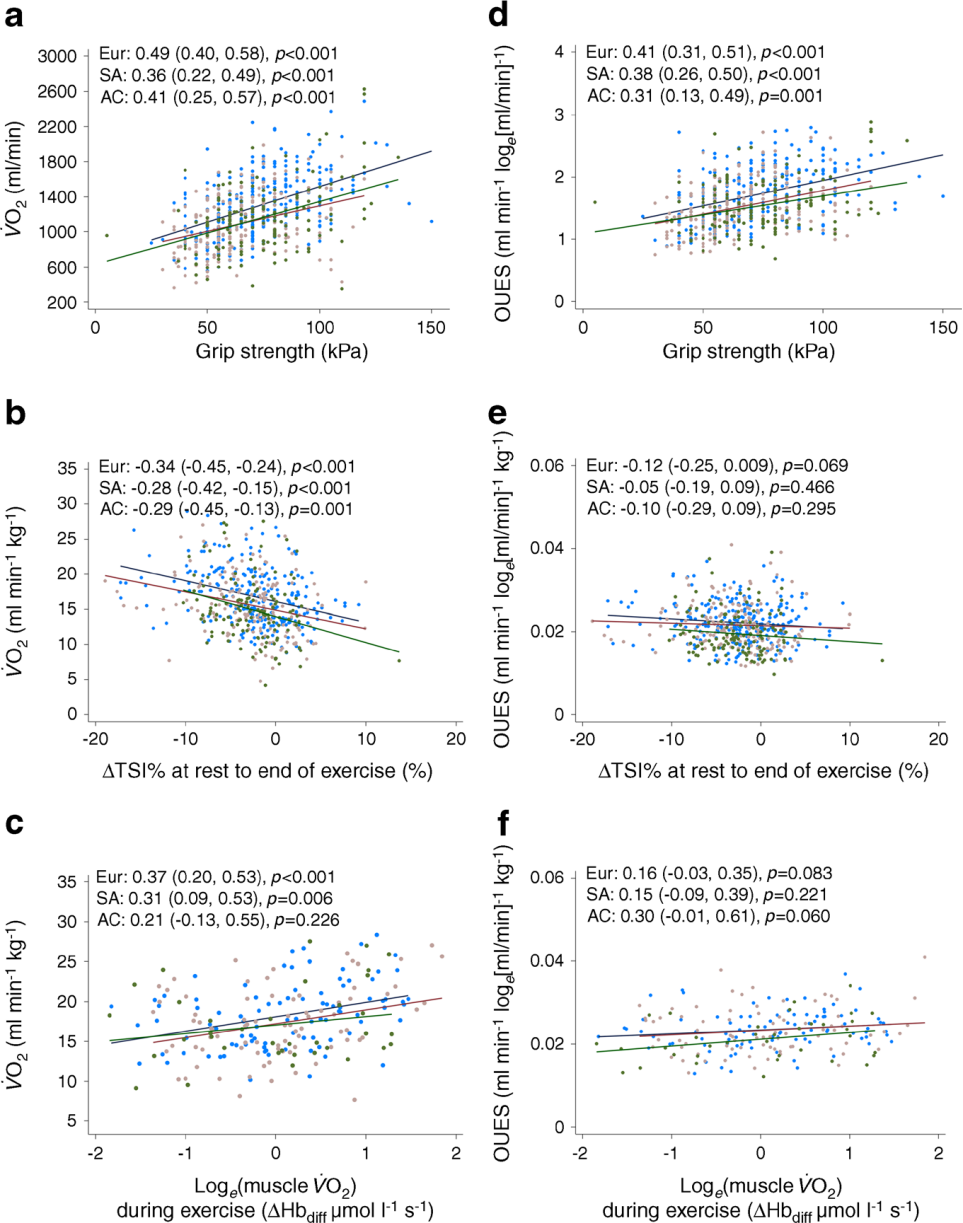
(**a**–**c**) Correlations between submaximal $\dot{V}{\mathrm{O}}_2$ and grip strength (**a**), muscle saturation (∆TSI%) (**b**) and muscle $\dot{V}{\mathrm{O}}_2$at peak exercise (**c**). (**d**–**f**) Correlations between OUES and grip strength (**d**), muscle saturation (**e**) muscle $\dot{V}{\mathrm{O}}_2$at peak exercise (**f**). Correlations are stratified by ethnicity and correlation coefficients (95% CI) for each ethnicity are given on each plot; the assocated *p* values indicate the significance of the association in each ethnic group. Blue, Europeans; brown, South Asians; green, African Caribbeans. AC, African Caribbean; Eur, European; SA, South Asian

Older adults were recruited from the Southall and Brent Revisited (SABRE) tri-ethnic cohort study of South Asian and African Caribbean migrants and European individuals resident in West London, UK. Participants were aged 40–69 years at initial recruitment in 1988 and initially, by design, more men than women were recruited in the European and South Asian subgroups [13]. However, at the follow-up visit (2015–2018; data from which are used in the present study), participants were invited to bring partners in order to achieve similar numbers of men and women. Participants and partners of participants who reported their ethnicity as European, South Asian or African Caribbean and had no contraindication to submaximal exercise testing based on American College of Sports Medicine (ACSM) guidelines, including angina or a recent cardiovascular event (myocardial infarction, stroke, TIA), an uncontrolled arrhythmia, uncontrolled arterial hypertension, severe aortic stenosis, severe symptoms of chronic obstructive pulmonary disease (COPD) and any orthopaedic impairment severely compromising exercise capacity [14], were included in this analysis. A subset of the full SABRE cohort underwent skeletal muscle measurements. Exclusion criteria included: intolerance of cuff inflation; an adipose tissue thickness (ATT) above the area of skeletal muscle undergoing measurement greater than 1.5 cm; broken skin around the area of skeletal muscle measurement; refusal to remove clothes to permit equipment for muscle measurements to be fitted; and unavailability of trained staff to carry out the measurement (criteria for exclusion are given in full in electronic supplemental material [ESM] Fig. 1).

All procedures were in accordance with the principles of the Helsinki declaration, all participants gave written informed consent and the study was approved by the National Research Ethics Service (NRES) Committee London—North Fulham.

### Anthropometrics and questionnaires

Height was measured barefoot using a stadiometer (seca 217; seca, Hamburg, Germany). Weight and fat-free mass (FFM) were measured using digital bio-impedance scales (BC-418; Tanita, IL, USA). Waist circumference was measured midway between the costal margins and the iliac crest using a tape measure. Hip circumference was measured at the nearest palpable point to the greater trochanters. Waist-to-hip ratio (WHR) was calculated. Diabetes was defined as self-reported physician diagnosis, or reported use of glucose-lowering medication or an elevated measurement of HbA_1c_ above the guideline cut-off value for diagnosis of type 2 diabetes (≥48 mmol/mol [>6.5%]) [15]. Information on physical activity, smoking, history of cardiovascular disease (CVD), hypertension and medication use were obtained by questionnaire. ATT overlaying the gastrocnemius was measured using an ultrasound device (Vivid I; GE, Boston, MA, USA) fitted with a high frequency transducer (12 L-RS; 6–13 MHz; GE).

### Blood samples

Non-fasting blood samples were obtained in the morning of the clinic visit following an earlier light breakfast. Haemoglobin was measured in fresh blood using an XE-2100 automated haematology analyser (Sysmex, Kobe, Japan). HbA_1c_ was measured in stored blood samples using an immunoassay (Cobas HbA_1c_ test) on the Cobas c311 automated analyser (Roche Diagnostics, Burgess Hill, UK). The assay was calibrated and quality controlled using the manufacturer’s reagents.

### Exercise capacity and whole-body

$\dot{\boldsymbol{V}}{\mathbf{O}}_{\mathbf{2}}$ Submaximal exercise capacity was measured using a 6 min stepper test (6MST), which has previously been validated in this age-group against the 6 min walk test [16]. The number of steps achieved during the test was used as a measure of self-paced exercise capacity. A portable expired gas analysis system including a Polar heart rate monitor (K4B2; COSMED, Rome, Italy) was used to measure breath-by-breath $\dot{V}{\mathrm{O}}_2$ and heart rate during the 6MST, and the highest $\dot{V}{\mathrm{O}}_2$ and heart rate of a rolling 60 s mean was determined. The oxygen uptake efficiency slope (OUES) was used as an index of maximum exercise performance and cardiopulmonary reserve based on submaximal exercise [17], and was calculated as the slope of $\dot{V}{\mathrm{O}}_2$(ml/min) vs the log of ventilation (log_*e*_VE; ml/min) across all data points measured during exercise [17].

### Grip strength

Grip strength was measured (in kPa) from the dominant hand using a Baseline hand-held pneumatic bulb dynamometer (3B Scientific, Hamburg, Germany). Three measurements were taken and the highest measurement achieved was classed as the maximum grip strength.

### Skeletal muscle measures: saturation during exercise, $\dot{\boldsymbol{V}}{\mathbf{O}}_{\mathbf{2}}$ (resting and post-exercise) and oxidative capacity

Skeletal muscle was assessed using NIRS to non-invasively assess changes in oxygenated and deoxygenated haemoglobin, and tissue saturation index (TSI%) with exercise. A NIRS device (Portamon; Artinis Medical Systems, Nijmegen, the Netherlands) [18, 19] was positioned on the lateral head of the gastrocnemius where the calf girth was greatest, orientation was standardised and the device was secured and covered completely using a neoprene sleeve.

Muscle $\dot{V}{\mathrm{O}}_2$ was measured at rest by inflating a rapid inflation cuff (Hokanson, SC10D/E20; PMS Instruments, Maidenhead, UK) placed on the thigh proximal to the location of NIRS measurement to induce complete arterial occlusion [20–22]. The cuff was deflated during exercise and inflated immediately following the submaximal exercise test in order to determine skeletal muscle post-exercise $\dot{V}{\mathrm{O}}_2$. Further occlusions were performed transiently (5–8 s intervals) throughout a 3 min recovery period to determine the kinetics of muscle $\dot{V}{\mathrm{O}}_2$ recovery, represented by a time constant (τ) [23]. τ has shown good reproducibility and agreement with the established ^31^P-MRS method of measuring skeletal muscle oxidative capacity [23, 24].

Analysis of NIRS data was conducted using custom written programs in MATLAB R2014a (MathWorks, Natick, MA, USA). The difference between TSI% at rest and at the end of exercise was calculated. Greater absolute reductions in this value for a given workload during exercise are thought to represent haemodynamic insufficiency [25]. This has been demonstrated during exercise when blood flow is impaired [26]. Resting and post-exercise muscle $\dot{V}{\mathrm{O}}_2$ is estimated from the slope of the difference between the oxygenated and deoxygenated haemoglobin signal (Hb_diff_) during each occlusion [27]; the units for these measurements are change in Hb_diff_ in μmol per litre per s (∆Hb_diff_ μmol l^−1^ /s^−1^). Using the difference signal as an index of $\dot{V}{\mathrm{O}}_2$ corrects for blood volume shifts during arterial occlusion [28]. Calculated in this way, more negative values represent higher muscle $\dot{V}{\mathrm{O}}_2$; therefore, in order to simplify interpretation, we re-expressed the values as positive numbers. The time constant, τ, was derived from a mono-exponential curve fitted to the slopes of recovery of muscle $\dot{V}{\mathrm{O}}_2$ measured post-exercise.

### Statistical analysis

Statistical analysis was carried out in STATA (StataCorp 2015, release 14; College Station, TX, USA). Categorical data are presented as *n*(%). Continuous descriptive data were examined for normality; normally distributed data are presented as mean±SD and skewed data are presented as median (interquartile range). Comparison of means was done using an unpaired Student’s *t* test for continuous data and *χ*^2^ test for categorical data. A Mann–Whitney *U* test was used for comparison of participant characteristics with skewed distribution. Multivariable linear regression was used to adjust associations for potential confounding factors; non-normally distributed data was appropriately transformed (as indicated in the text). The effect of ethnicity on outcome measures included a basic adjustment for age and sex to account for possible sampling bias. Further adjustment for the following possible mediators was also performed: type 2 diabetes, HbA_1c_, haemoglobin, CVD, habitual physical activity, FFM, WHR, hypertension and β-blocker use. Multiple imputation with 30 datasets was used to account for missing covariates, and complete case analyses were conducted to check for similarity of trends (ESM Methods and ESM Table 1 provide full information on missing data and analysis methods). Where muscle measurements also depended on the work achieved during exercise (∆TSI% and post-exercise muscle $\dot{V}{\mathrm{O}}_2$), adjustment was also made for the number of steps completed during the 6MST. Marginal mean differences with 95% CIs between the three ethnic groups were calculated. Correlations between exercise capacity (submaximal: $\dot{V}{\mathrm{O}}_2$; estimated maximal: OUES) and measures of muscle function were examined, stratified by ethnicity and, using Pearson’s correlation coefficients with 95% CIs, estimated by bootstrapping. Statistical significance was assigned if *p* < 0.05.

## Results

### Participants

In total, 988 participants attended the SABRE study clinic. Of these, 280 participants were excluded from this analysis as they were ineligible or unable to undertake the exercise test; largely, exclusion was for contraindication to exercise testing (ESM Fig. 1). In this study, 708 participants (mean age, 73 ± 7 years; male, *n* = 395 [56%]) undertook a 6MST and a forearm grip strength test. African Caribbeans were slightly younger than the other two ethnic groups, and there were more men in the European and South Asian groups, reflecting initial recruitment patterns. Type 2 diabetes prevalence was twofold greater in African Caribbeans and 2.5-fold greater in South Asians than in Europeans. BMI was higher, but WHR lower, in African Caribbeans compared with both Europeans and South Asians (Table 1).

**Table 1:** Participant characteristics by ethnicity

Characteristic	Ethnicity			*p* value^a^		
	Eur	SA	AC	Eur–SA	Eur–AC	SA–AC
Male						
*n*	311	232	165			
*n*(%)	192 (62)	140 (60)	63 (38)	0.742	<0.001	<0.001
Age (years)						
*n*	311	232	165			
mean±SD	74.0 ± 5.9	73.1 ± 6.0	70.7 ± 8.0	0.094	<0.001	0.001
BMI (kg/m^2^)						
*n*	311	232	165			
mean±SD	27.8 ± 4.2	26.6 ± 3.8	29.5 ± 4.7	<0.001	<0.001	<0.001
FFM (kg)						
*n*	304	228	164			
mean±SD	53.4 ± 9.7	47.7 ± 8.6	51.8 ± 9.1	<0.001	0.088	<0.001
WHR						
*n*	310	232	161			
mean±SD	0.96 ± 0.08	0.97 ± 0.09	0.92 ± 0.09	0.086	<0.001	<0.001
ATT (cm)						
*n*	254	192	112			
mean±SD	0.72 ± 0.39	0.76 ± 0.40	0.76 ± 0.44	0.324	0.369	0.904
T2D						
*n*	311	232	165			
*n*(%)	42 (14)	77 (33)	47 (28)	<0.001	<0.001	0.319
HbA_1c_						
*n*	306	228	161			
mmol/mol, median (IQR)	37 (34–41)	41 (38–47)	38 (33–44)	<0.001	0.516	<0.001
%, median (IQR)	5.5 (5.3–5.9)	5.9 (5.6–6.5)	5.6 (5.2–6.2)	<0.001	0.516	<0.001
Hb (mmol/mol)						
*n*	310	230	161			
mean±SD	145 ± 13	138 ± 14	136 ± 12	<0.001	<0.001	0.143
Resting HR (bpm)						
*n*	310	231	165			
mean±SD	68 ± 11	65 ± 11	68 ± 12	<0.001	0.862	0.009
β-blocker use						
*n*	311	232	165			
*n*(%)	37 (12)	56 (24)	17 (10)	<0.001	0.601	<0.001
Physical activity (MJ/week)						
*n*	265	162	118			
median (IQR)	5.3 (3.4–7.7)	5.7 (3.5–7.8)	4.8 (2.9–6.7)	0.723	0.283	0.188
Smoking category						
*n*	293	186	138			
Never, *n*(%)	131 (45)	165 (89)	95 (69)	<0.001	<0.001	<0.001
Ex smoker, *n*(%)	153 (52)	20 (11)	36 (26)	<0.001	<0.001	<0.001
Current smoker, *n*(%)	9 (3)	1 (0.5)	7 (5)	<0.001	<0.001	<0.001
HTN present						
*n*	307	228	161			
*n*(%)	143 (47)	153 (67)	105 (65)	<0.001	<0.001	0.698
CVD						
*n*	311	232	165			
*n*(%)	38 (12)	47 (20)	18 (11)	0.010	0.680	0.013

Categorical data are presented as *n*(%); normally distributed continuous data are presented as mean±SD; skewed data are shown as median (interquartile range)

^a^Pairwise comparison

AC, African Caribbean; bpm, beats per min; Eur, European; Hb, venous haemoglobin; HR, heart rate; HTN, hypertension; IQR, interquartile range; SA, South Asian, T2D, type 2 diabetes

### Ethnic differences in submaximal exercise capacity

After adjustment for age and sex, South Asians completed fewer steps than Europeans and African Caribbeans and achieved a lower peak heart rate. Additionally, submaximal exercise capacity, in terms of $\dot{V}{\mathrm{O}}_2$ml min^−1^ kg^−1^, was markedly lower in both South Asians and African Caribbeans compared with Europeans (Table 2). Although these differences in $\dot{V}{\mathrm{O}}_2$and heart rate persisted with adjustment for either type 2 diabetes or HbA_1c_, the difference in heart rate between Europeans and South Asians no longer reached the criteria for assignment of statistical significance. Differences in $\dot{V}{\mathrm{O}}_2$ between Europeans and African Caribbeans persisted with adjustment for type 2 diabetes and other possible mediators: physical activity, CVD, WHR, smoking status, haemoglobin, hypertension and β-blocker use; while the differences in $\dot{V}{\mathrm{O}}_2$ and heart rate between Europeans and South Asians was further attenuated following these adjustments (Table 2). OUES was lower in South Asians compared with both Europeans and African Caribbeans (Table 2) and this difference was essentially unaffected by adjustment for type 2 diabetes or HbA_1c_ and only modestly attenuated by inclusion of other potential mediators.

**Table 2:** Ethnic differences in exercise capacity with multivariable adjustment

Variable	Ethnicity			*p* value^b^		
	Adj. mean (95% CI)	β coefficient (95% CI) vs Eur^a^				
	Eur	SA	AC	Eur–SA	Eur–AC	SA–AC
Steps (*n*)	*n* = 311	*n* = 232	*n* = 165			
Adj. for age, sex	218 (210, 226)	−34 (−46, −22)	−9 (−22, 5.1)	<0.001	0.218	0.001
Adj. for age, sex, T2D	215 (208, 223)	−28 (−41, −16)	−4 (−18, 9)	<0.001	0.537	0.001
Adj. for age, sex, HbA_1c_	216 (208, 223)	−28 (−40, −15)	−7 (−21, 7)	<0.001	0.312	0.005
Adj. for age, sex, T2D, PA, CVD, WHR, smoking, Hb, HTN, BB	214 (206, 222)	−25 (−38, −11)	−4 (−18, 11)	<0.001	0.616	0.004
$\dot{V}{\mathrm{O}}_2$ (ml min^−1^ kg^−1^)	*n* = 277	*n* = 208	*n* = 143			
Adj. for age, sex	16.6 (16.2, 17.1)	−1.2 (−1.9, −0.4)	−1.7 (−2.5, −0.8)	0.002	<0.001	0.264
Adj. for age, sex, T2D	16.4 (16.0, 16.9)	−0.8 (−1.5, −0.1)	−1.4 (−2.2, −0.5)	0.027	0.001	0.215
Adj. for age, sex, HbA_1c_	16.5 (16.1, 17.0)	−0.9 (−1.7, −0.2)	−1.6 (−2.4, −0.8)	0.014	<0.001	0.142
Adj. for age, sex, T2D, PA, CVD, WHR, smoking, Hb, HTN, BB	16.3 (15.8, 16.8)	−0.6 (−1.4, 0.2)	−1.2 (−2.1, −0.4)	0.167	0.005	0.129
Peak HR (bpm)	*n* = 275	*n* = 206	*n* = 144			
Adj. for age, sex	124 (121, 127)	−4(−9, −0.3)	5 (−0.2, 9)	0.037	0.061	<0.001
Adj. for age, sex, T2D	124 (121, 127)	−4 (−8, 0.1)	5 (−0.02, 10)	0.057	0.051	<0.001
Adj. for age, sex, HbA_1c_	124 (121, 127)	−4 (−8, 0.3)	5 (−0.1, 10)	0.065	0.054	0.001
Adj. for age, sex, T2D, PA, CVD, WHR, smoking, Hb, HTN, BB	124 (121, 127)	−3 (−8, 2)	4 (−1, 9)	0.196	0.149	0.008
OUES (ml min^−1^ log_*e*_[ml/min]^−1^)	*n* = 275	*n* = 207	*n* = 141			
Adj. for age, sex	1.71 (1.67, 1.75)	−0.22 (−0.28, −0.15)	−0.11 (−0.19, −0.04)	<0.001	0.004	0.008
Adj. for age, sex, T2D	1.71 (1.67, 1.75)	−0.21 (−0.28, −0.15)	−0.11 (−0.18, −0.03)	<0.001	0.006	0.008
Adj. for age, sex, HbA_1c_	1.71 (1.67, 1.76)	−0.22 (−0.29, −0.16)	−0.11 (−0.19, −0.04)	<0.001	0.003	0.006
Adj. for age, sex, T2D, PA, CVD, WHR, smoking, Hb, HTN, BB	1.68 (1.63, 1.72)	−0.15 (−0.23, −0.08)	−0.05 (−0.13, 0.02)	<0.001	0.179	0.015

^a^β coefficients (95% CI) indicate differences vs Europeans

^b^Pairwise comparison

AC, African Caribbean; Adj., adjusted; BB, β-blockers; bpm, beats per min; Eur, European; Hb, venous haemoglobin; HR, heart rate; HTN, hypertension; PA, physical activity; SA, South Asian; T2D, type 2 diabetes

### Ethnic differences in grip strength and skeletal muscle measures

Grip strength was lowest in South Asians, highest in African Caribbeans and intermediate in Europeans (Table 3). These differences were attenuated for South Asians vs Europeans, but not for African Caribbeans vs South Asians or Europeans, after adjustment for potential mediators: type 2 diabetes, FFM, physical activity level, WHR, CVD, smoking, haemoglobin, hypertension and β-blocker use (Table 3).

**Table 3:** Ethnic differences in skeletal muscle with multivariable adjustment

Variable	Ethnicity			*p* value^b^		
	Adj. mean (95% CI)	β coefficient (95% CI) vs Eur^a^				
	Eur	SA	AC	Eur–SA	Eur–AC	SA–AC
Grip strength (kPa)	*n* = 311	*n* = 232	*n* = 165			
Adj. for age, sex	72 (70, 73)	−9 (−12, −6)	6 (3, 9)	<0.001	<0.001	<0.001
Adj. for age, sex, FFM, T2D	70 (68, 72)	−4 (−7, −0.7)	6 (3, 9)	0.016	<0.001	<0.001
Adj. for age, sex, FFM, HbA_1c_	70 (68, 72)	−4 (−7, −0.6)	6 (3, 9)	0.017	<0.001	<0.001
Adj. for age, sex, FFM, T2D, PA, CVD, WHR, smoking, Hb, HTN, BB	69 (68, 71)	−3 (−6, 0.5)	7 (3, 10)	0.100	<0.001	<0.001
∆TSI(%)	*n* = 257	*n* = 179	*n* = 139			
Adj. for age, sex	−2.3 (−2.8, −1.8)	−0.5 (−1.3, 0.3)	−0.4 (−1.3, 0.5)	0.245	0.380	0.872
Adj. for age, sex, $\dot{V}{\mathrm{O}}_2$	−2.1 (−2.6, −1.6)	−0.9 (−1.7, −0.1)	−0.9 (−1.8, −0.02)	0.024	0.046	0.970
Adj. for age, sex, $\dot{V}{\mathrm{O}}_2$, T2D	−2.1 (−2.7, −1.6)	−0.8 (−1.6, 0.05)	−0.8 (−1.7, 0.1)	0.065	0.088	0.977
Adj. for age, sex, $\dot{V}{\mathrm{O}}_2$_,_ HbA_1c_	−2.1 (−2.6, −1.6)	−0.8 (−1.6, −0.001)	−0.9 (−1.8, 0.01)	0.051	0.052	0.886
Adj. for age, sex, $\dot{V}{\mathrm{O}}_2$, T2D, PA, CVD, WHR, smoking, Hb, HTN, BB	−2.2 (−2.8, −1.7)	−0.5 (−1.4, 0.4)	−0.8 (−1.7, 0.2)	0.274	0.109	0.599
Resting muscle $\dot{V}{\mathrm{O}}_2$ (ΔHb_diff_ μmol l^−1^ /s^−1^)	*n* = 126	*n* = 100	*n* = 57			
Adj. for age, sex	0.30 (0.27, 0.34)	−0.04 (−0.10, 0.01)	0.02 (−0.04, 0.09)	0.105	0.502	0.057
Adj. for age, sex, T2D	0.31 (0.27, 0.34)	−0.05 (−0.10, 0.01)	0.02 (−0.05, 0.09)	0.086	0.577	0.057
Post-exercise muscle $\dot{V}{\mathrm{O}}_2$ (ΔHb_diff_ μmol l^−1^ /s^−1^)	*n* = 97	*n* = 82	*n* = 40			
Adj. for age, sex	1.47 (1.24, 1.69)	−0.14 (−0.47, 0.19)	0.10 (−0.33, 0.53)	0.402	0.638	0.279
Adj. for age, sex, $\dot{V}{\mathrm{O}}_2$	1.39 (1.17, 1.60)	0.02 (−0.30, 0.34)	0.23 (−0.18, 0.64)	0.914	0.277	0.327
Adj. for age, sex, $\dot{V}{\mathrm{O}}_2$, T2D	1.40 (1.18, 1.61)	0.001 (−0.32, 0.32)	0.21 (−0.21, 0.62)	0.995	0.325	0.334
Adj. for age, sex, $\dot{V}{\mathrm{O}}_2$, HbA_1c_	1.40 (1.18, 1.61)	−0.01 (−0.33, 0.31)	0.23 (−0.18, 0.63)	0.939	0.280	0.269
Adj. for age, sex, $\dot{V}{\mathrm{O}}_2$, T2D, CVD, smoking, Hb, HTN, BB	1.36 (1.12, 1.60)	0.08 (−0.30, 0.46)	0.27 (−0.18, 0.72)	0.691	0.236	0.391
τ (s)	*n* = 80	*n* = 74	*n* = 31			
Adj. for age, sex	43 (38, 48)	11 (3, 18)	7 (−3, 17)	0.006	0.164	0.498
Adj. for age, sex, T2D	44 (38, 49)	10 (2, 17)	6 (−4, 16)	0.016	0.240	0.513
Adj. for age, sex, HbA_1c_	43 (38, 48)	11 (3, 19)	7 (−3, 17)	0.008	0.165	0.528
Adj. for age, sex, T2D, PA, CVD, WHR, smoking, Hb, HTN, BB	43 (37, 49)	11 (1, 20)	7 (−4, 17)	0.026	0.239	0.976

^a^β coefficients (95% CI) indicate differences vs Europeans

^b^Pairwise comparison

AC, African Caribbean; Adj., adjusted; BB, β-blockers; bpm, beats per min; Eur, European; HTN, hypertension; PA, physical activity; SA, South Asian; T2D, type 2 diabetes

South Asians had the greatest decrease in TSI% after exercise and Europeans the smallest. The difference between Europeans and South Asians persisted after adjustment for age, sex, whole-body $\dot{V}{\mathrm{O}}_2$ and type 2 diabetes or HbA_1c_ (Table 3) and the effect sizes were little altered after adjustment for type 2 diabetes and other mediators (age, sex, $\dot{V}{\mathrm{O}}_2$, physical activity level, CVD, WHR, smoking, haemoglobin, hypertension and β-blocker use; Table 3). Resting muscle $\dot{V}{\mathrm{O}}_2$ in the gastrocnemius was lowest in South Asians and highest African Caribbeans, although 95% CIs for the three ethnic groups overlapped (Table 3). Post-exercise muscle $\dot{V}{\mathrm{O}}_2$ (adjusted for whole-body $\dot{V}{\mathrm{O}}_2$) was highest African Caribbeans, although 95% CIs for the three ethnic groups also overlapped for this muscle measure (Table 3). Estimated maximum oxidative capacity during recovery from exercise (indicated by the time constant, τ, during recovery from exercise) was poorer (longer τ) in South Asians compared with Europeans (*p* = 0.006; Table 3). Adjusting models for all potential mediators (age, sex, type 2 diabetes, physical activity levels, CVD, WHR, smoking, haemoglobin, hypertension and β-blocker use) had little effect on the ethnic differences in oxidative capacity between Europeans and South Asians (*p* = 0.026; Table 3).

### Correlations between submaximal whole-body $\dot{\boldsymbol{V}}{\mathbf{O}}_{\mathbf{2}}$ and skeletal muscle measures

Stronger grip was associated with higher submaximal $\dot{V}{\mathrm{O}}_2$ (correlation coefficient [bootstrapped 95% CI]: 0.44 [0.37, 0.51]; *p* < 0.001). Stratification by ethnicity showed some evidence for stronger associations in Europeans than South Asians, with African Caribbeans having intermediate values (correlation coefficient [bootstrapped 95% CI]: Europeans, 0.49 [0.40, 0.58]; African Caribbeans, 0.41 [0.25, 0.57]; South Asians, 0.36 [0.22, 0.49]; *p* < 0.001 for all; Fig. 1a).

Greater declines in TSI% (greater negative values) were associated with higher submaximal $\dot{V}{\mathrm{O}}_2$ (correlation coefficient [bootstrapped 95% CI]: −0.30 [−0.37, −0.23]; *p* < 0.001). When stratified by ethnicity, there was no evidence of ethnic differences in the strength of association (Fig. 1b).

Higher post-exercise muscle $\dot{V}{\mathrm{O}}_2$was associated with higher $\dot{V}{\mathrm{O}}_2$(correlation coefficient [bootstrapped 95% CI]: 0.31 [0.19, 0.44]; *p* < 0.001). When stratified by ethnicity, there was some evidence for stronger associations in Europeans and South Asians compared with African Caribbeans (correlation coefficient [bootstrapped 95% CI]: Europeans, 0.37 [0.20, 0.53], *p* < 0.001; African Caribbeans, 0.21 (−0.13, 0.55), *p* = 0.226; South Asians, 0.31 (0.09, 0.53), *p* = 0.006; Fig. 1c).

Resting muscle $\dot{V}{\mathrm{O}}_2$and estimated maximum oxidative capacity (τ) were not associated with submaximal $\dot{V}{\mathrm{O}}_2$ (correlation coefficient [bootstrapped 95% CI]: resting muscle $\dot{V}{\mathrm{O}}_2$, 0.07 [−0.05, 0.20], *p* = 0.243; estimated maximum oxidative capacity, −0.04 [−0.12, 0.20], *p* = 0.636). Ethnicity did not modify these associations (ESM Fig. 2a, b).

### Correlations between OUES and skeletal muscle measures

Stronger grip was positively associated with higher OUES (correlation coefficient [bootstrapped 95% CI]: 0.38 (0.31, 0.46); *p* < 0.001). Stratification by ethnicity showed no evidence for stronger associations in Europeans and African Caribbeans than South Asians (Fig. 1d).

Greater changes in TSI% were associated with higher OUES (correlation coefficient [bootstrapped 95% CI]: −0.09 (−0.18, −0.009); *p* = 0.029). When stratified by ethnicity, there was no evidence for an ethnic difference in the strength of association (Fig. 1e).

Higher post-exercise muscle $\dot{V}{\mathrm{O}}_2$was associated with higher OUES (correlation coefficient [bootstrapped 95% CI]: 0.19 [0.05, 0.32]; *p* = 0.008). When stratified by ethnicity, there was weak evidence of a stronger association in African Caribbeans (*p* = 0.060) compared with Europeans (*p* = 0.083) and South Asians (*p* = 0.221) (Fig. 1f).

Resting muscle $\dot{V}{\mathrm{O}}_2$and oxidative capacity (τ) were not associated with OUES (correlation coefficient [bootstrapped 95% CI]: resting muscle $\dot{V}{\mathrm{O}}_2$, 0.08 (−0.05, 0.21), *p* = 0.233; oxidative capacity, −0.10 (−0.27, 0.06), *p* = 0.227). These associations did not differ by ethnicity (see ESM Fig. 2).

## Discussion

We show that South Asians and African Caribbeans have lower submaximal exercise capacity and that this is not accounted for by the excess diabetes prevalence in these two ethnic groups. Grip strength, tissue saturation, post-exercise muscle $\dot{V}{\mathrm{O}}_2$and maximum capacity for oxidative fuel consumption were all poorer in South Asians compared with both Europeans and African Caribbeans; again, this was not accounted for by excess type 2 diabetes prevalence. In contrast, despite also sharing elevated diabetes prevalence with South Asians, African Caribbeans had a significantly greater grip strength than Europeans.

Our findings are aligned with previous reports of poorer overall aerobic capacity in South Asians and African Caribbeans compared with Europeans [4, 8, 11, 29]. The role of type 2 diabetes in accounting for these ethnic differences has not previously been explored. Strikingly, we show no impact of adjustment for type 2 diabetes on these ethnic differences. This was also the case when we adjusted for hyperglycaemia (HbA_1c_) in place of type 2 diabetes, thus, providing evidence that this finding is not due to misclassification of diabetes status by ethnicity.

As well as adjustment for type 2 diabetes, additional adjustment for other potential mediators (age, sex, physical activity levels, CVD, WHR, smoking, haemoglobin, hypertension and β-blocker use) only minimally attenuated the European–African Caribbean differences in $\dot{V}{\mathrm{O}}_2$ and had negligible effect on differences in number of steps completed. One potential explanation for these differences is that exercise or physical activity habits differ by ethnicity in older adults or throughout the preceding life course. While we adjusted for current physical activity levels, we did not account for early-to-midlife activity and a limitation of our study is that we used self-reported measures, which do not fully reflect objectively measured physical activity [30]. However, Ghouri et al previously described lower moderate-to-vigorous physical activity (MVPA), measured using wearable devices, in South Asians, and this difference in MVPA did not account for the lower $\dot{V}{\mathrm{O}}_2$ in this population [4].

Greater changes in muscle saturation (∆TSI%) represent the balance between supply (the capacity of the cardiovascular system to deliver oxygen via the vasculature) and demand (the workload imposed). Thus, greater decreases in TSI% (more negative values) for equivalent work imply impaired oxygen delivery, probably as a result of poorer perfusion [25]. ∆TSI% was greatest in South Asians after adjusting for $\dot{V}{\mathrm{O}}_2$, suggesting an impairment in muscle perfusion in South Asians, even during submaximal exercise. The difference in TSI% between South Asians and Europeans persisted after adjustment for type 2 diabetes or HbA_1c_ (though significance was lost) and, although the difference was slightly attenuated by adjusting for other mediators alongside $\dot{V}{\mathrm{O}}_2$ and type 2 diabetes (age, sex, physical activity level, CVD, WHR, smoking, haemoglobin, hypertension and β-blocker use), the β coefficient for ∆TSI% in South Asians vs Europeans changed by only 0.3, from −0.8 (*p* = 0.051) to −0.5 (*p* = 0.274). These data suggest that underlying vascular dysfunction contributes to impaired exercise capacity in South Asians, consistent with previous observations of impaired macro- and microvascular function in South Asian individuals [31, 32].

In South Asians, compared with Europeans, in vivo measured skeletal muscle oxidative capacity was reduced. Findings from previous studies of skeletal muscle oxidative capacity in South Asians and Europeans are inconsistent [8–10]. Compared with Europeans, the expression of oxidative and lipid metabolism genes is commonly reported as equivalent in South Asians, existing alongside lower capacity for muscle fat oxidation [8] and, in some studies, lower mitochondrial:nuclear DNA ratio [9], while others report enhanced mitochondrial DNA copy number [10]. The in vivo method described here cannot provide the molecular insights provided by muscle biopsy analyses. Nevertheless, the simple marker of oxidative capacity used in our study provides information about the overall maximal capacity for cellular oxidative fuel metabolism measured within a physiological environment, capturing all interrelated systemic pathways. Our study provides evidence for an overall impairment in oxidative capacity in the skeletal muscle of South Asians.

Oxidative capacity was also lower in African Caribbeans than Europeans, but the 95% CIs were wide and the observations were indicative of no difference between the groups. Previous findings have reported lower oxidative capacity in skeletal muscle biopsies from young-to-middle aged African-American women, as compared with Americans of European descent [12, 33]. In our study, there was little convincing evidence that post-exercise muscle $\dot{V}{\mathrm{O}}_2$ and resting muscle $\dot{V}{\mathrm{O}}_2$ differed between African Caribbeans and Europeans. A limitation of our study is that fewer African Caribbeans participated at the follow-up visit from which data used in this study were obtained and, of these, fewer participants undertook the detailed skeletal muscle NIRS measurements. This limited the precision of our estimates in African Caribbeans and may have contributed to our failure to observe some differences reported in previous studies [34].

A strength of the present study is that it was based on a sample that is reasonably representative of the major ethnic groups in the UK [13]. However, this analysis was observational and cross-sectional and, therefore, causal inferences cannot be made. Based on other studies, the question of whether impaired oxidative capacity is a cause or consequence of type 2 diabetes remains contentious [35, 36], yet we show that type 2 diabetes does not mediate any of the ethnic differences observed in the exercise capacity or muscle measures in our study. We did not find an association between oxidative capacity and submaximal exercise capacity. Further work is necessary to determine the precise molecular mechanisms responsible for this impairment.

Grip strength was lower in South Asians compared with Europeans, in keeping with previous findings [5]. African Caribbeans were stronger than Europeans (in terms of grip strength), which is in line with some, but not all, previous findings [5, 37]. Adjustment for type 2 diabetes (or HbA_1c_) and FFM only marginally attenuated differences in strength. This was surprising because epidemiological data suggests type 2 diabetes is associated with low strength [5, 38] and that this association is substantially stronger in South Asians and black African men compared with Europeans [5]. We cannot explain the differences between study findings but speculate that the effect of ageing on muscle strength could vary with ethnicity; given our study participants were older, a slower rate of decline in African Caribbeans would contribute to strength differences in older adults. Further adjustment for potential mediators largely attenuated the European–South Asian difference in grip strength but could not explain the greater strength in African Caribbeans. As discussed above, due to the inherent limitations of self-reported physical activity level, we cannot conclusively establish that the differences in grip strength were not due to differences in physical activity or exercise habits. While we included adjustment for the most prevalent comorbidities (type 2 diabetes and CVD), it is also possible that differences in muscle function and submaximal exercise capacity could be explained by factors that we have not accounted for. Grip strength was positively associated with higher submaximal $\dot{V}{\mathrm{O}}_2$, consistent with muscle power influencing submaximal exercise capacity [39]. There was some evidence for a weaker correlation between $\dot{V}{\mathrm{O}}_2$ and grip strength in South Asians compared with Europeans. However, the correlation between grip strength and OUES, a marker of maximal cardiorespiratory function, was similar by ethnicity.

Previous study sample populations have generally included young or middle aged, active participants free from comorbidities. Typically, these participants undertake maximal exercise tests to assess aerobic capacity ($\dot{V}{\mathrm{O}}_2$ at volitional exhaustion). While cardiac factors are largely considered to govern peak $\dot{V}{\mathrm{O}}_2$ in healthy individuals [40], the determinants of submaximal exercise capacity in older individuals are less well defined. Given the ageing population and known age-related decline in mitochondrial energetics [41, 42], this study included older adults (>65 years old), assessed submaximal exercise capacity and examined overall mitochondrial oxidative capacity. These methodological differences could account for the lack of effect of type 2 diabetes in multivariable models. Higher cardiorespiratory fitness has been shown in non-Hispanic white compared with black Americans [11] and in Europeans compared with South Asians [4, 29]; in these studies, as in ours, ethnic differences could not be explained by differences in anthropometrics, smoking or physical activity levels. These prior studies enrolled relatively young (20–49 years old) participants in whom it less likely that differences were influenced by comorbidities, providing evidence for the existence of an undescribed determinant, or multiple determinants, of ethnic differences in fitness.

In addition to the strengths and limitations discussed above, ATT at the site of NIRS measurement is known to influence measurements by increasing photon scattering [21]. Ultrasound measurements of ATT above the NIRS-interrogated region showed no differences by ethnicity, so it is unlikely that this will have introduced major bias to our analyses.

Future work, investigating sub-clinical measures (for example, measures of micro- and macrovascular dysfunction) or novel biomarkers as potential mediators of the ethnic differences observed in this study would be interesting. Further investigation of the impact of nutritional status on age-related sarcopenia as an explanation of these differences should be considered. Conducting similar studies in younger individuals would provide complementary insight into ethnic differences in exercise capacity and muscle function at earlier stages in the life course. The use of objective wearable activity monitors to investigate the effect of patterns of physical activity/exercise habits, such as frequency, duration, intensity and type of activity, on ethnic differences in exercise capacity is also warranted.

### Conclusions

South Asians have reduced submaximal exercise capacity, muscle strength and oxidative capacity compared with Europeans. We have been unable to fully explain these differences by higher rates of type 2 diabetes, hyperglycaemia, differences in whole-body metabolic health, or other risk factors, such as low physical activity level, smoking and the presence of CVD or hypertension. Grip strength, ∆TSI% and peak muscle $\dot{V}{\mathrm{O}}_2$ are associated with exercise capacity; these associations may differ by ethnicity. The discordance between lower exercise capacity and greater muscle strength, despite higher rates of type 2 diabetes, in African Caribbeans is an intriguing area for future work.

## Electronic supplementary material

ESM (PDF 1.25 mb)

## Data Availability

The datasets generated and/or analysed during the current study are available from the corresponding author on reasonable request.

## Abbreviations

ATT: Adipose tissue thickness
CVD: Cardiovascular disease
FFM: Fat-free mass
Hb_diff_: Difference between the oxygenated and deoxygenated haemoglobin signal
6MST: 6 min stepper test
MVPA: Moderate-to-vigorous physical activity
NIRS: Near infrared spectroscopy
OUES: Oxygen uptake efficiency slope
^31^P-MRS: ^31^P-magnetic resonance spectroscopy
SABRE: Southall and Brent Revisited (study)
TSI: Tissue saturation index
