# Supplementary material for: Type 2 diabetes does not account for ethnic differences in exercise capacity or skeletal muscle function in older adults
Source: Diabetologia. 2019 Dec 9;63(3):624–35. doi: 10.1007/s00125-019-05055-w (PMC6997264; doi:10.1007/s00125-019-05055-w)
Supplement: Supplementary file 1 — (PDF 1.25 mb) [file 125_2019_5055_MOESM1_ESM.pdf]

Electronic supplemental material (ESM)

ESM Methods: Statistical methods for imputation of missing covariates

Of all participants included in these analyses (n=708), missing values for covariates ranged from 0 to 163 (0 to 23%). The variables with most missing values were physical activity level (n=163, 23%) and smoking status (n=91, 13%), this was due to poorly completed or incomplete questionnaires. Information about presence of cardiovascular disease (CVD), hypertension (HTN) and use of β-blockers (BB) was missing due to incomplete questionnaires. Values for venous blood glycated haemoglobin (HbA1c) and haemoglobin (Hb) concentrations were missing because the blood sample could not be processed. Waist-hip ratio (WHR) was missing if the participant declined to remove or adjust clothing to allow the hip or waist measurements to be made according to the protocol. Values for fat-free mass (FFM) were missing in 12 individuals in whom bio-impedance scales could not be used due to a pacing device (n=5) or a technical problem with the scales (n=7). We assumed data were missing at random and covariates were imputed using multiple imputation by chained equations (30 imputations were performed). Complete case analyses were also conducted to check each model. Outcome variables were not imputed and covariates age, sex and T2DM were complete in all models. For each outcome in tables 2 and 3 in the main manuscript, the number of missing values are provided in table 1a. The imputation model and auxiliary variables are listed in table 1b.

ESM Table 1a number of missing values imputed for covariates

Outcome	CC (n)	Covariate/mediator (missing values; n(%))								
		PA	smoking	CVD	HbA1c	WHR	FFM	Hb	HTN	BB
**Steps**	708	163(23)	91(13)	23(3)	13(2)	5(1)	0	7(1)	12(2)	4(1)
**VO_2**	628	134(21)	82(13)	20(3)	12(2)	3(1)	12(2)	6(1)	10(2)	3(1)
**Peak HR**	625	134(21)	81(13)	20(3)	12(2)	4(1)	0	6(1)	10(2)	3(1)
**OUES**	623	134(22)	82(13)	20(3)	12(2)	3(1)	12(2)	6(1)	10(2)	3(1)
**Grip**	708	163(23)	91(13)	23(3)	13(2)	5(1)	12(2)	7(1)	12(2)	4(1)
**ΔTSI**	575	123(21)	65(11)	19(3)	11(2)	5(1)	8(1)	7(1)	8(1)	1(1)
**τ**	185	37(20)	28(15)	2(1)	1(1)	0	3(2)	0	0	0

Abbreviations: BB; beta blocker use, CC; complete case, CVD; cardiovascular disease, FFM; fat free mass, Hb; venous haemoglobin, HbA1c; glycated haemoglobin, HR; heart rate, HTN; hypertension, OUES; oxygen uptake efficiency slope, PA; physical activity, TSI; tissue saturation index, $\dot{V}O_2$; oxygen consumption, WHR; waist-hip ratio.

ESM Table 1b Imputation model and auxiliary variables

Outcome	Imputation model and auxiliary variables
**Steps**	Ethnicity, sex, age, T2DM, HbA1c, weight, height, weight, grip strength
**VO_2**	Ethnicity, sex, age, T2DM, HbA1c, height, grip strength, steps completed
**Peak HR**	Ethnicity, sex, age, T2DM, HbA1c, weight, height, weight, grip strength, steps completed
**OUES**	Ethnicity, sex, age, T2DM, HbA1c, weight, height, weight, grip strength, steps completed
**Grip**	Ethnicity, sex, age, T2DM, HbA1c, weight, height, weight, steps completed
**ΔTSI**	Ethnicity, sex, age, T2DM, HbA1c, weight, height, weight, grip strength, steps completed
**τ**	Ethnicity, sex, age, T2DM, HbA1c, weight, height, weight, grip strength, steps completed, VO_2, resting & post-exercise muscle VO_2, Hb, WHR, BB, HTN

Abbreviations: BB; beta blocker use, Hb; venous haemoglobin, HR; heart rate, HTN; hypertension, HbA1c; glycated haemoglobin, OUES; oxygen uptake efficiency slope, TSI; tissue saturation index, $\dot{V}O_2$; oxygen consumption, WHR; waist-hip ratio.

ESM Fig. 1a Flow chart of recruitment, inclusion and analysis of 6 minute stepper test

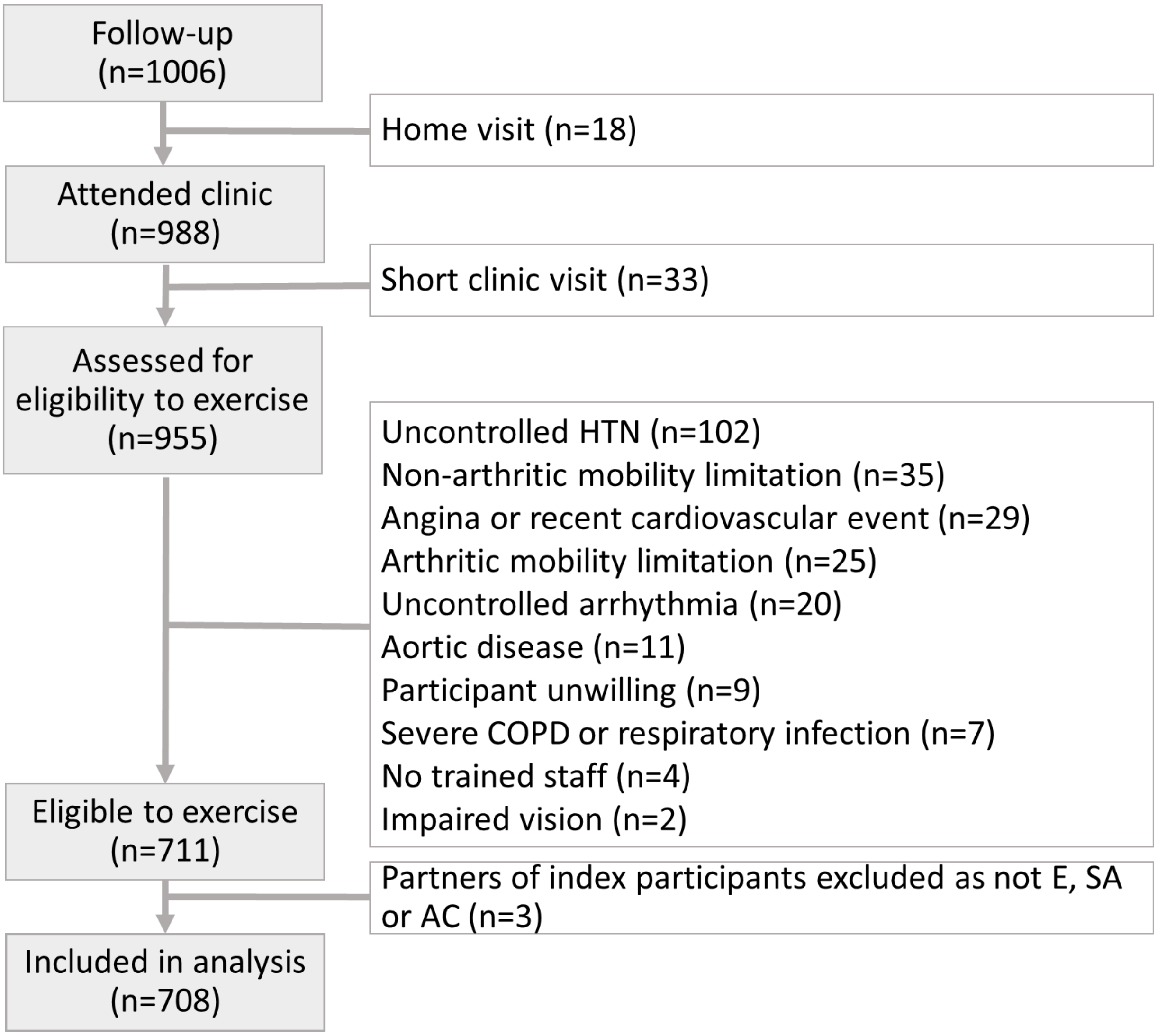

Abbreviations: AC; African-Caribbean, E; European, SA; South Asian, COPD; chronic obstructive pulmonary disease, HTN; hypertension.

ESM Fig. 1b Flow chart of recruitment, inclusion and analysis of near infrared spectroscopy measurements of skeletal muscle.

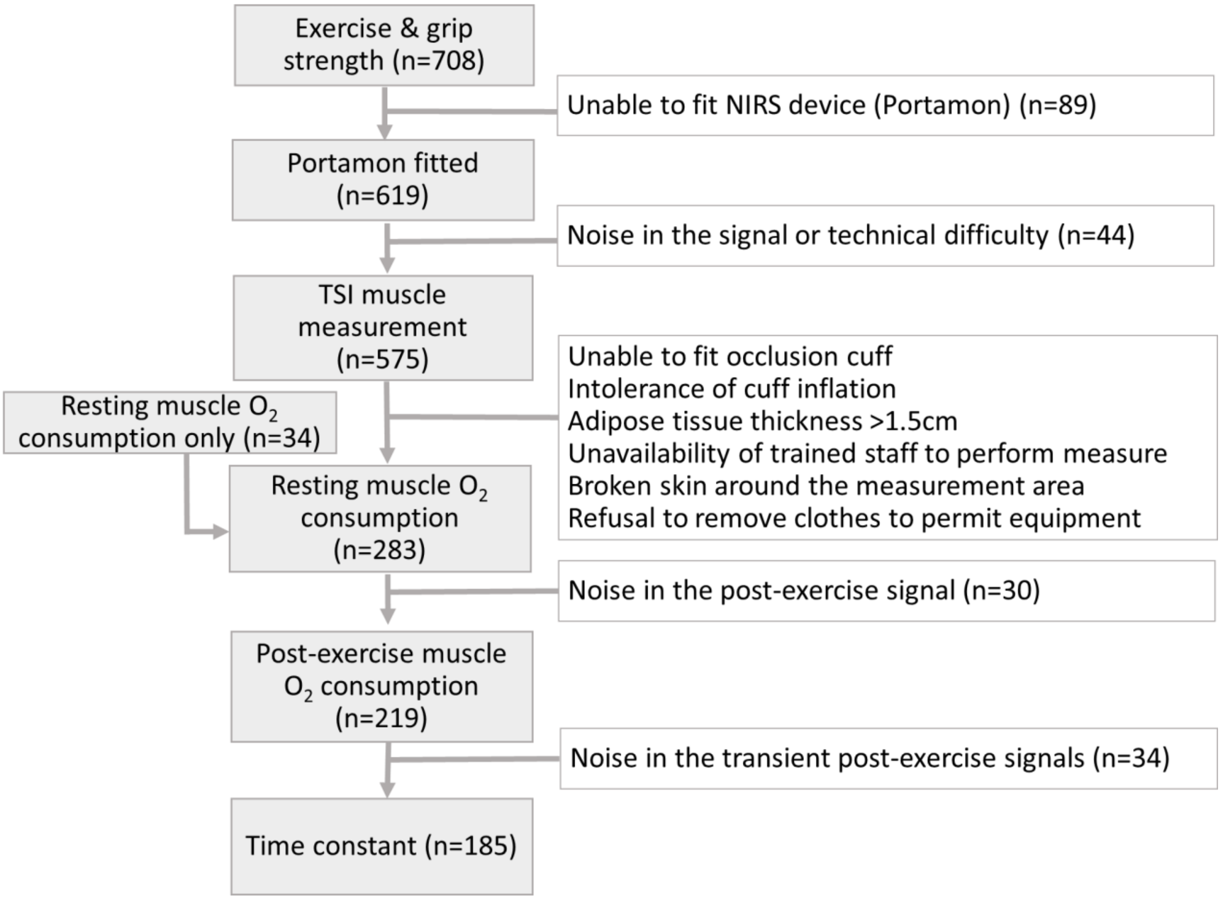

Abbreviations: TSI; tissue saturation index. A sub-set of 34 participants undertook resting arterial occlusions but were excluded from exercise due to contra-indications given above. This group are highlighted as '*Resting muscle O_2 consumption only (n=34)*' in this chart.

ESM Fig. 2 additional correlation analysis

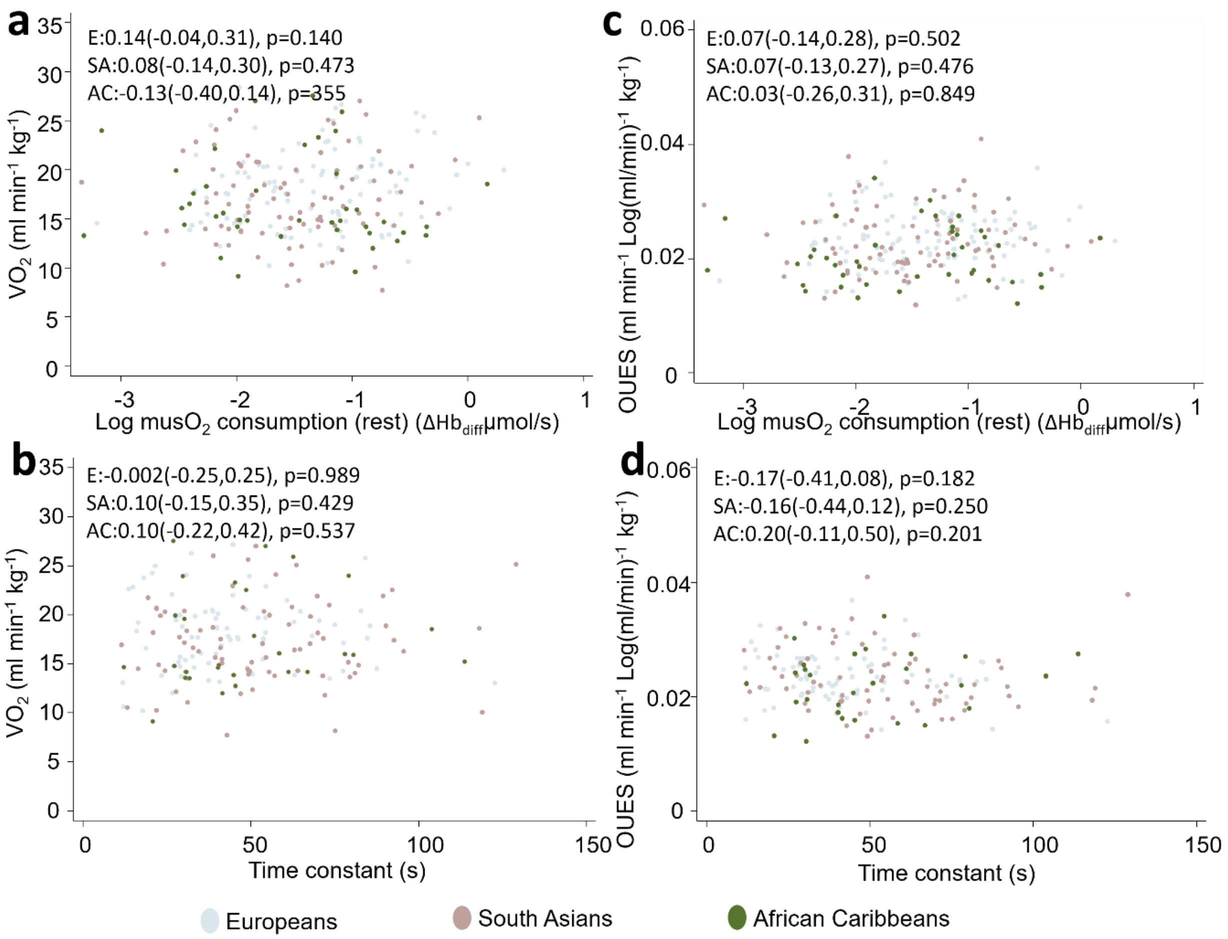

Resting muscle oxygen consumption was not associated with sub-maximal VO2 or oxygen uptake efficiency slope (OUES). Correlations are shown, stratified by ethnicity. Bootstrapped correlation coefficients (95%CI) and p values are given on each plot for correlations between whole-body $\dot{V}O_2$ with (a) resting muscle oxygen consumption and (b) oxidative capacity (τ) and oxygen uptake efficiency slope (OUES) with (c) resting muscle oxygen consumption and (d) oxidative capacity (τ), stratified by ethnicity. Abbreviations: AC; African-Caribbean, E; European, SA; South Asian.

StudyID

Questionnaire Part 1a

SABRE STUDY

Diabetes and heart disease research study

Thank you for taking the time to fill in this questionnaire.

In this study we are following up people who took part in a health survey in West London between 1989 and 1991 and between 2008 and 2011.
At this new follow-up we will also invite the partners of the original study group to join in.

We want to continue to study the differences in health that occur in people from different ethnic origins. The research will build on the findings from 1988 – 1991 and help us to find out whether and why some groups of people are healthy and why some are more at risk of diabetes, heart disease, strokes and other serious illnesses.

If you would like some help with filling in the questionnaire, one of the study team will be happy to help you to fill it in when you visit the clinic or to go through it with you by telephone – please do contact us on 020 7679 9471 or email: sabre@ucl.ac.uk

You can visit our website at www.sabrestudy.org

General instructions

Please answer all the questions, except where the instructions indicate otherwise.

Most questions can be answered by writing a number or by putting a mark in the box like this:

☒ One answer ☐ Another answer

Please print any text answers in capitals LIKE THIS

If you would like some help with filling in the questionnaire, one of the study team will be happy to help you to fill it in by telephone or when you visit the clinic

Please return the questionnaire to us in the reply paid envelope provided.

All information that you give will be treated as strictly confidential.

SABRE Study-Freepost
UCL Institute of Cardiovascular Science
Gower Street
London WC1E 6BT

1.1 Please enter today's date

		/			/				

(day) (month) (year)

1.2 Your year of birth

1	9		

1.3 Your sex

☐ Male ☐ Female

1.4 Which one of the following best describes you at present

☐ Single
☐ Married or living with partner
☐ Widowed
☐ Divorced or separated
☐ I have a partner, but we don’t live together
☐ Other, please state

1.5 May we send you some more questionnaires about your lifestyle, physical functioning and disability, family history of illness and other topics which affect health?

☐ Yes, I am willing for the SABRE study team to send me questionnaires in the future
☐ No, I would prefer not to receive further questionnaires

(If you agree, we expect to send 2 more questionnaires within the next 2-3 months and may send similar questionnaires on a yearly basis. You will receive a £5 gift voucher for each questionnaire completed)

1.6 We would like to access information from your medical records and link this data with other health-related records.

For example, linking your study records with information from your GP records or from hospital admissions or national database records (such as heart attacks or cancer). This information will be treated with the utmost care and attention to confidentiality. *(See section 'Your medical and health-related records' under 'Taking part in the SABRE Study' in the participant information booklet)*

If you DO NOT want us to have access to your records please tick the box ☐

Section 2: Ethnicity and country of birth

2.1 To which of these ethnic groups do you feel you belong?

☐ I do not wish to answer this question

Black or Black British

☐ Black Caribbean

☐ Black African

☐ Any other Black background (Please specify) ________

Asian or Asian British

☐ Bangladeshi

☐ East African Asian

☐ Indian

☐ Pakistani

☐ Sri Lankan

☐ Tamil

☐ Chinese

☐ Any other Asian background (Please specify) ________

White or White British

☐ English

☐ Irish

☐ Scottish

☐ Welsh

☐ Eastern European

☐ Any other White background (Please specify) ________

Mixed

☐ Mixed Asian (Please specify) ________

☐ Mixed Black (Please specify) ________

☐ Mixed White (Please specify) ________

☐ Any other mixed background (Please specify) ________

2.2 In which country were you born?

- ☐ England
- ☐ Scotland
- ☐ Northern Ireland
- ☐ Wales
- ☐ Eire
- ☐ India
- ☐ Bangladesh
- ☐ Kenya
- ☐ Tanzania
- ☐ China
- ☐ Jamaica
- ☐ Barbados
- ☐ Pakistan
- ☐ Sri Lanka
- ☐ Uganda
- ☐ Malaysia
- ☐ Trinidad
- ☐ Guyana
- ☐ Other (Please specify) [ ]

2.3 If you were not born in England, how old were you when you first moved here?

[ ][ ] years

2.4 Were both your parents born in the same country as you?

- ☐ Yes
- ☐ No →

What country was your father born in? [ ]

What country was your mother born in? [ ]

Section 3: Your health

3.1 Compared with others your age, would you say that your health over the last 12 months has been:

- ☐ Very good
- ☐ Good
- ☐ Average
- ☐ Poor
- ☐ Very Poor

3.2 Thinking about your own life and personal circumstances, how satisfied are you with your life as a whole? Please circle a number.

1	2	3	4	5	6	7	8	9	10
Not at all satisfied									Extremely Satisfied

3.3 Have you ever had a heart attack (coronary thrombosis or myocardial infarction (MI)) which was confirmed by a doctor?

- ☐ No
- ☐ Yes → In what year did this first happen? ☐☐☐☐

Have you had any more heart attacks (confirmed by a doctor) since then?

- ☐ No
- ☐ Yes → Year of most recent heart attack: ☐☐☐☐

3.4 Have you ever had angina (chest pain from the heart) which was confirmed by a doctor)?

- ☐ No
- ☐ Yes → In what year did this first happen? ☐☐☐☐

And when did you last have angina?

- ☐ Within the past month
- ☐ 1-12 Months Ago
- ☐ More than a year ago

3.5 Do you have heart failure which has been confirmed by a doctor? (symptoms may include shortness of breath or swelling of your ankles or feet)

☐ No
☐ Yes → In what year did this first happen? [ ][ ][ ][ ]

3.6 Have you ever had narrowing or hardening of the arteries in the leg which has been confirmed by a doctor? (This could be called claudication or peripheral arterial disease or peripheral vascular disease)

☐ No
☐ Yes → In what year did this first happen? [ ][ ][ ][ ]

3.7 Have you ever had an operation called a coronary artery bypass graft (or CABG) for heart trouble/ angina?

☐ No
☐ Yes → In what year did this first happen? [ ][ ][ ][ ]

Have you had any more CABG operations since then?

☐ No
☐ Yes → Year of most recent CABG: [ ][ ][ ][ ]

3.8 Have you ever had an operation called an angioplasty where tubes (stents) or balloons were placed in the coronary arteries for heart trouble?

☐ No
☐ Yes → In what year did this first happen? [ ][ ][ ][ ]

Have you had any more angioplasty operations since then?

☐ No
☐ Yes → Year of most recent angioplasty: [ ][ ][ ][ ]

3.9 Have you ever had an operation called an angiogram to look at the coronary arteries (in your heart)?

☐ No
☐ Yes → Please give year of most recent angiogram: | | | | |

3.10 Have you ever had high blood pressure which was confirmed by a doctor?

☐ No
☐ Yes → In what year were you first told? | | | | |

are you now receiving any tablets or medicines to help control your blood pressure?

☐ No
☐ Yes

3.11 Do you have diabetes which was confirmed by a doctor?

☐ No
☐ Yes → In what year were you first told? | | | | |

are you now receiving any tablets to help control your diabetes?

☐ No
☐ Yes

are you now receiving any injections to help control your diabetes?

☐ No
☐ Yes

3.12 Have you ever had a stroke or TIA (transient ischaemic attack or mini-stroke) which was confirmed by a doctor?

☐ No

☐ Yes → Please give year of first stroke/TIA [| | |]

How long did the symptoms last?

☐ Less than 24 Hours

☐ 24 Hours or more

Have you had any more strokes or TIAs (confirmed by a doctor) since then?

☐ No

☐ Yes → Year of most recent stroke/TIA [| | |]

Have you made a complete recovery from your stroke(s)?

☐ No

☐ Yes

Because of your stroke(s), do you need help carrying out your usual activities?

☐ No

☐ Yes

3.13 Have you ever had cancer which was confirmed by a doctor?

☐ No

☐ Yes → Which year did this first happen? [| | |]

Do you still have cancer?

☐ No

☐ Yes

Which parts of your body are or were affected?

[ ]

3.14 Have you ever been told by a doctor that you have any of the following problems?

	No	Yes
Kidney Disease	☐	☐
Lung problems such as chronic bronchitis or emphysema	☐	☐
Asthma	☐	☐
Arthritis	☐	☐
Osteoporosis	☐	☐

3.15 Please list any other serious health problems not yet mentioned:

3.16 Have you been admitted to hospital during the past year?

☐ No ☐ Yes

If yes, please give some details for each admission to hospital

Month (1-12)	Reason you were admitted (brief)

Section 4: Medication

Are you currently taken any regular medication?

☐ No ☐ Yes

Please list below the names of **ALL** medications that you take regularly. Make sure to include all medications including drops, inhalers, vitamins, ointments. Please also list any medicines which you buy yourself.

Name of medication	Reason for taking (if known)	Year Started (if known)	Is this medication prescribed? Yes	No
			☐	☐
			☐	☐
			☐	☐
			☐	☐
			☐	☐
			☐	☐
			☐	☐
			☐	☐
			☐	☐
			☐	☐
			☐	☐
			☐	☐

Thank you very much for taking the time to fill in this questionnaire.

We very much appreciate your help

Please bring the questionnaire with you when you come to our clinic or return it to us in the reply paid envelope

SABRE Study-Freepost
UCL Institute of Cardiovascular Science
Gower Street
London WC1E 6BT

Tel: 020 7679 9471

5374268

StudyID

UCL

sabre

Southall and Brent Revisited

Diabetes & Heart Disease Research Study

Questionnaire Part 1b

SABRE STUDY

Diabetes and heart disease research study

Thank you for taking the time to fill in this questionnaire.

In this study we are following up people who took part in a health survey in West London between 1989 and 1991 and between 2008 and 2011.
At this new follow-up we will also invite the partners of the original study group to join in.

We want to continue to study the differences in health that occur in people from different ethnic origins. The research will build on the findings from 1988 – 1991 and help us to find out whether and why some groups of people are healthy and why some are more at risk of diabetes, heart disease, strokes and other serious illnesses.

If you would like some help with filling in the questionnaire, one of the study team will be happy to help you to fill it in when you visit the clinic or to go through it with you by telephone – please do contact us on 020 7679 9471 or email: sabre@ucl.ac.uk

You can visit our website at www.sabrestudy.org.uk

General instructions

Please answer all the questions, except where the instructions indicate otherwise.

Most questions can be answered by writing a number or by putting a mark in the box like this:

☒ One answer ☐ Another answer

Please print any text answers in capitals LIKE THIS

If you would like some help with filling in the questionnaire, one of the study team will be happy to help you to fill it in by telephone or when you visit the clinic

Please bring the questionnaire with you when you come for your clinic visit or return it to us in the reply paid envelope provided.

All information that you give will be treated as strictly confidential.

SABRE Study-Freepost
UCL Institute of Cardiovascular Science
Gower Street
London WC1E 6BT

1.1 Please enter today's date

		/			/				

(day) (month) (year)

1.2 Your **year** of birth

1	9		

Please continue to the next page

Section 5: Sleep

5.1 Do you have difficulty falling asleep?

☐ No ☐ Yes

5.2 Do you usually wake up too early?

☐ No ☐ Yes

5.3 Do you usually still feel tired when you wake up in the morning?

☐ No ☐ Yes

5.4 In the past year, have you at any time been woken at night by an attack of breathlessness?

☐ No ☐ Yes

5.5 How often do you snore at night? (If you are not sure, please ask someone who is likely to know)

☐ Never snore
☐ Occasionally snore
☐ Often snore
☐ Almost always snore
☐ Don’t know

5.6 How many hours do you usually sleep at night?

☐☐ hours

5.7 Have you ever been told that you hold your breath during sleep? (stop breathing for at least 10 seconds)

☐ No ☐ Yes

Section 6: Tiredness, breathlessness and other symptoms

6.1 Do you regularly feel tired when carrying out usual daily activities?

☐ No ☐ Yes

6.2 Do you regularly have any swelling in your feet, ankles, legs or abdomen?

(Please tick all that apply)

☐ No swelling

☐ Swelling in feet

☐ Swelling in ankles

☐ Swelling in legs

☐ Swelling in abdomen

6.3 Do you ever get breathless when you are lying down?

☐ No ☐ Yes

6.4 Do you ever get short of breath walking with other people of your own age on level ground?

☐ No

☐ Yes

☐ I am unable to walk

6.5 On walking uphill or upstairs, do you get more breathless than other people of your own age?

☐ No

☐ Yes

☐ I am unable to walk

6.6 Do you ever have to stop walking because of breathlessness?

☐ No

☐ Yes

☐ I am unable to walk

6.7 Have you ever been told by a doctor that you have had any of the following:

	No	Yes
Atrial Fibrillation	☐	☐
Deep vein thrombosis (clot in a deep leg vein)	☐	☐
Pulmonary embolism (clot on the lung)	☐	☐

Section 7: Women Only

MEN- please go to section 8

7.1 Have you ever used birth control pills or had birth control injections?

☐ No

☐ Yes →

7.2 How old were you when you began using birth control pills/injections?

[ ][ ] years of age

7.3 For how many years did you use birth control pills/injections?

[ ][ ] years

7.4 Have you ever used hormone replacements (HRT) to help you with the menopause?

☐ No

☐ Yes →

For how long? [ ][ ] years

7.5 Are you still taking HRT?

☐ No → At what age did you stop? [ ][ ] years of age

☐ Yes

7.6 Have you ever been pregnant?

☐ No

☐ Yes →

7.7 Did you ever have a miscarriage or stillbirth?

☐ No

☐ Yes → please state how many ☐☐

7.8a How many live-born babies have you had? ☐☐

7.8b. What were their birth weights (if applicable)?

1	☐☐	pounds	☐☐	ounces
2	☐☐	pounds	☐☐	ounces
3	☐☐	pounds	☐☐	ounces
4	☐☐	pounds	☐☐	ounces

7.9 Did you ever have high blood pressure during pregnancy?

☐ No

☐ Yes

7.10 Did you ever have diabetes during pregnancy?

☐ No

☐ Yes

Section 8: Smoking

8.1 Have you ever smoked cigarettes?

☐ No

☐ Yes →

8.2 How old were you when you started smoking regularly?

[|]

8.3 Do you smoke cigarettes at present?

☐ No →

☐ Yes →

8.4 How old were you when you stopped smoking regularly?

[|] years of age

8.5 When you smoked, how many cigarettes did you usually smoke in a day?

[|] cigarettes

8.6 How often do you smoke cigarettes?

☐ Daily

☐ 4-5 days a week

☐ Only occasionally

8.7 About how many cigarettes do you usually smoke each day that you smoke?

[|] cigarettes

or if tobacco

[|] ounces

Section 9: Alcohol

9.1 Have you ever had a drink containing alcohol in your life?

☐ No

☐ Yes, but given up completely

☐ Yes →

9.2 How often do you normally have an alcoholic drink?

☐ Daily

☐ 4-5 days a week

☐ Once or twice a week

☐ Once or twice a month

☐ Special occasions only

9.3 What is your preferred drink?

☐ Wine

☐ Beer

☐ Spirits

☐ Combination of beers, wines or spirits

☐ Low alcohol drinks

☐ Other ________ (please specify)

9.4 If one drink is half a pint of beer/lager/cider,
or a single whisky, gin, brandy, vodka or other spirit
or one glass of wine (one bottle of wine contains 6 glasses)

How much do you usually drink on the days when you drink alcohol?

☐ More than 6 drinks

☐ 5-6 drinks

☐ 3-4 drinks

☐ 1-2 drinks

9.5 How many alcoholic drinks do you have during an average week? ☐☐

9.6 Is the alcohol which you drink usually taken (tick all that apply)

☐ Before meals

☐ With meals

☐ After meals

☐ Separate from meals

Section 10: Diet and weight

10.1 How much do you weigh now?

[ ][ ] stone [ ][ ] pounds or [ ][ ][ ] kg

10.2 Are you on any of the following diets?

- ☐ Weight reduction diet
- ☐ Diabetic diet
- ☐ Cholesterol-lowering diet
- ☐ Fasting or abstaining for religious reasons
- ☐ Other diet [ ] (please specify)
- ☐ Not on a diet

10.3 Which of the following do you think best describes your weight?

- ☐ Underweight
- ☐ About the right weight
- ☐ A little overweight
- ☐ Very overweight

10.4 In total, how many teaspoons of sugar do you usually use each day in drinks like tea and coffee or on food at a table (e.g. breakfast cereal)?

- ☐ None
- ☐ 1-2 teaspoons
- ☐ 3-5 teaspoons
- ☐ 6-10 teaspoons
- ☐ 11-20 teaspoons
- ☐ More than 20 teaspoons

10.5 What type of milk do you usually use?

- ☐ None
- ☐ Whole milk (full fat/full cream) - blue top
- ☐ Semi-skimmed-green top
- ☐ Skimmed - red top
- ☐ Soya
- ☐ Other [ ] (please specify)

10.6 In a typical week during the past month or so, how often did you eat each of the following foods?

Meat, eggs and dairy	Rarely or never	Less than 1 a week	Once a week	2-3 times a week	4-6 times a week	1-2 times a day	3-4 times a day	5+ a day
Eggs	☐	☐	☐	☐	☐	☐	☐	☐
Milk, butter or cheese	☐	☐	☐	☐	☐	☐	☐	☐
Fish or seafood	☐	☐	☐	☐	☐	☐	☐	☐
Chicken	☐	☐	☐	☐	☐	☐	☐	☐
Lamb	☐	☐	☐	☐	☐	☐	☐	☐
Beef (inc. burgers)	☐	☐	☐	☐	☐	☐	☐	☐
Pork, ham or bacon	☐	☐	☐	☐	☐	☐	☐	☐
Processed meats such as salami, corned beef, luncheon meat etc	☐	☐	☐	☐	☐	☐	☐	☐

Fruit and vegetables	Rarely or never	Less than 1 a week	Once a week	2-3 times a week	4-6 times a week	1-2 times a day	3-4 times a day	5+ a day
Fruit	☐	☐	☐	☐	☐	☐	☐	☐
Green vegetables (tinned frozen/fresh)	☐	☐	☐	☐	☐	☐	☐	☐
Boiled, mashed or jacket potatoes	☐	☐	☐	☐	☐	☐	☐	☐
Fried or roast potatoes or fried chips	☐	☐	☐	☐	☐	☐	☐	☐
Oven-cooked chips	☐	☐	☐	☐	☐	☐	☐	☐

Bread, rice, pasta, pulses	Rarely or never	Less than 1 a week	Once a week	2-3 times a week	4-6 times a week	1-2 times a day	3-4 times a day	5+ a day
Bread/chapattis/parathas/puris /nan/pittas etc	☐	☐	☐	☐	☐	☐	☐	☐
Boiled rice	☐	☐	☐	☐	☐	☐	☐	☐
Fried rice	☐	☐	☐	☐	☐	☐	☐	☐
Pasta (spaghetti etc)	☐	☐	☐	☐	☐	☐	☐	☐
Pulses (such as lentils, kidney beans, soya beans etc)	☐	☐	☐	☐	☐	☐	☐	☐

10.6 Continued

Other	Rarely or never	Less than 1 a week	Once a week	2-3 times a week	4-6 times a week	1-2 times a day	3-4 times a day	5+ a day
Breakfast cereals	☐	☐	☐	☐	☐	☐	☐	☐
Savoury snacks (e.g. crisps or corn snacks, Bombay mix, etc)	☐	☐	☐	☐	☐	☐	☐	☐
Samosas, pakoras, spring rolls	☐	☐	☐	☐	☐	☐	☐	☐
Sausage rolls, pasties, pork pies	☐	☐	☐	☐	☐	☐	☐	☐
Ready meals(take-away, chip shop, supermarket chilled meals etc	☐	☐	☐	☐	☐	☐	☐	☐

10.7 In a typical week what type of fat or oil did you use when preparing food?

Please tick one or more boxes in each column where applicable.

Fat/oils	Baking	Frying	Spreading	Salads
Butter/Ghee	☐	☐	☐	☐
Low far spread	☐	☐	☐	☐
Margarine soft tub	☐	☐	☐	☐
Hard margarine - brick	☐	☐	☐	☐
Other vegetable oil	☐	☐	☐	☐
Palm / coconut oil	☐	☐	☐	☐
Lard	☐	☐	☐	☐
Other ______	☐	☐	☐	☐

10.8 What is your favourite meal?

______

10.9 How often do you have your favourite meal?

☐ Hardly ever
☐ Once a month or less
☐ Once in 2 weeks
☐ 1-3 times a week
☐ 4-7 times a week
☐ Once a day or more

Section 11: Physical activity

11.1 On a typical day for you, how often do you do the following activities:
(please tick one box for each activity)

	Never	Seldom	Sometimes	Often	Always
Do you sit	☐	☐	☐	☐	☐
Do you stand	☐	☐	☐	☐	☐
Do you walk	☐	☐	☐	☐	☐
Do you lift heavy loads	☐	☐	☐	☐	☐

11.2 How many miles do you walk on an average weekday?

☐ Less than half a mile
☐ Between half a mile and one mile
☐ 1-3 miles
☐ 4 miles or more

Half a mile is about the distance from Southall railway station to the Broadway or the distance between Wembley Central and Wembley Stadium railway stations. Oxford Street is about one and a quarter miles end to end.

11.3 How many miles do you walk on an average day at the weekend?

☐ Less than half a mile
☐ Between half a mile and one mile
☐ 1-3 miles
☐ 4 miles or more

11.4 How fast do you usually walk?

☐ Slow
☐ Medium
☐ Fast

11.5 Do you ride a bicycle regularly (at least once a week)?

☐ No
☐ Yes →

11.6 How many miles do you cycle during an average week?

11.7 Do you play any sport (or take other recreational exercise such as going to the gym, swimming or dancing)?

- ☐ No
- ☐ Yes →

11.8 Which sport or other exercise do you play/do most frequently?

11.9 How many hours a week do you play this sport or take this exercise?

- ☐ Less than 1 hour/week
- ☐ 1-2 hours/week
- ☐ 3-4 hours/week
- ☐ 5 or more hours/week

11.10 How many months a year do you play this sport or take this exercise?

- ☐ Less than one month a year
- ☐ 1-3 months a year
- ☐ 4-5 months a year
- ☐ 6 months or more a year

11.11 How many hours a day do you sit and watch television or use a computer (on a typical day)?

- ☐ Less than 2 hours
- ☐ 2-3 hours
- ☐ 4-8 hours
- ☐ More than 8 hours a day.

Section 12: Activites of daily living

12.1 What is the furthest you can walk on your own without stopping and without discomfort?

- ☐ 200 yards (metres) or more
- ☐ More than a few steps but less than 200 yards (metres)
- ☐ Only a few steps

12.2 Can you walk up and down a flight of 12 stairs without resting?

- ☐ Yes
- ☐ Only if I hold on and take a rest
- ☐ Not at all

12.3 Can you when standing, bend down and pick up a shoe from the floor?

- ☐ No
- ☐ Yes

12.4 Please indicate if you have difficulty doing any of the following activities

	No difficulty	Some difficulty	Unable to do or need some help
Reaching or extending your arms above shoulder level	☐	☐	☐
Pulling or pushing large objects like a living room chair	☐	☐	☐
Walking across a room	☐	☐	☐
Getting in and out of a chair on your own	☐	☐	☐
Dressing or undressing yourself on your own	☐	☐	☐
Bathing or showering	☐	☐	☐
Feeding yourself , including cutting food	☐	☐	☐
Getting to and using the toilet on your own	☐	☐	☐
Lifting and carrying something as heavy as 10 lbs (for example, a bag of groceries)	☐	☐	☐
Shopping for personal items such as toilet items or medicines by yourself	☐	☐	☐
Doing light housework such as washing up	☐	☐	☐
Preparing your own meals by yourself	☐	☐	☐
Using the telephone by yourself	☐	☐	☐
Taking medications by yourself	☐	☐	☐
Managing money (for example, paying bills)	☐	☐	☐
Using public transport on your own	☐	☐	☐
Driving a car on your own	☐	☐	☐
Gripping with hands (for example, opening a jam jar)	☐	☐	☐

Section 13: Memory and falls

In the past year:

13.1 How often did you have trouble remembering things?

☐ Never

☐ Rarely

☐ Sometimes

☐ Often

13.2

	No	Yes
Did you have more trouble than usual remembering recent events?	☐	☐
Did you have more trouble than usual remembering a short list of items such as a shopping list?	☐	☐
Did you have trouble remembering things from one second to the next?	☐	☐
Did you have any difficulty in understanding or following spoken instruction?	☐	☐
Did you have more trouble than usual following a group conversation or a plot on TV due to your memory?	☐	☐
Did you have trouble finding your way around familiar streets or places?	☐	☐
Did you have trouble getting things organised/organising your day?	☐	☐
Did you have trouble concentrating on things e.g reading a book?	☐	☐

13.3 Have you had spells of dizziness, loss of balance or a sensation of spinning in the past year?

☐ No

☐ Yes

13.4 At the present time are you afraid that you may fall over?

☐ Very fearful

☐ Somewhat fearful

☐ Not fearful

13.5 Have you had a fall in the past year?

☐ No

☐ Yes →

13.6 How many falls in the past year?

13.7 Did you receive medical attention for any of these falls?

☐ No

☐ Yes

13.8 Did you suffer any of the following as a result of a fall in the past year? *(tick all that apply)*

☐ Cuts and bruises

☐ Damage to muscle or ligament

☐ Broken or fractured hip bone

☐ Broken or fractured wrist

☐ Other broken or fractured bone

Section 14: Your health overall

Please indicate which statements best describe your health <u>TODAY</u>

Mobility
- ☐ I have no problems in walking about
- ☐ I have some problems in walking about
- ☐ I am confined to bed/cannot walk at all

Self care
- ☐ I have no problems with self-care
- ☐ I have some problems washing or dressing myself
- ☐ I am unable to wash or dress myself

Usual activities
- ☐ I have no problems with performing my usual activities
- ☐ I have some problems with performing my usual activities
- ☐ I am unable to perform my usual activities

Pain/discomfort
- ☐ I have no pain or discomfort
- ☐ I have moderate pain or discomfort
- ☐ I have extreme pain or discomfort

Anxiety/depression
- ☐ I am not anxious or depressed
- ☐ I am moderately anxious or depressed
- ☐ I am extremely anxious or depressed

Health scale

Thermometer

We have drawn a health scale rather like a thermometer on which perfect health is 100 and 0 is the worst state you can imagine.

Please put a cross (**X**) on the scale below to reflect how good or bad your health is today

Worst imaginable health | Best imaginable health

0 10 20 30 40 50 60 70 80 90 100

EQ-5D-3L © 1990 EuroQol Group

Section 15: The way you have been feeling recently

15.1 Are you basically satisfied with your life?
☐ No
☐ Yes

15.2 Have you dropped many of your activities and interests?
☐ No
☐ Yes

15.3 Do you feel that your life is empty?
☐ No
☐ Yes

15.4 Are you afraid that something bad is going to happen?
☐ No
☐ Yes

15.5 Do you feel happy most of the time?
☐ No
☐ Yes

15.6 Do you often feel helpless?
☐ No
☐ Yes

15.7 Do you often feel that you have more problems with memory than most?
☐ No
☐ Yes

15.8 Do you feel full of energy?
☐ No
☐ Yes

15.9 Do you feel that your situation is hopeless?

☐ No

☐ Yes

15.10 Do you think that most people are better off then you are?

☐ No

☐ Yes

Section 16: Home, work and social circumstances

Research has shown that peoples' health may be affected by their personal, financial and social circumstances - this is why we are asking the questions in this section.

16.1 Are you at present:

- ☐ Living alone
- ☐ Living with a partner or spouse
- ☐ Living with other family members
- ☐ Living with other people

16.2 How many people live in your household? ☐☐

16.3 Your accommodation: are you at present:

- ☐ An owner occupier
- ☐ Renting from the local authority or a housing association
- ☐ Renting privately
- ☐ Living in a residential home
- ☐ Living in a nursing home
- ☐ Living in sheltered accommodation
- ☐ Other ________ (please specify)

16.4		No	Yes
	a. Do you have a car or van available for your own use?	☐	☐
	b. Do you drive yourself?	☐	☐
	c. Have you given up driving?	☐	☐

16.5 at what age did you give up driving?

☐☐ years of age

16.6 Why did you give up driving?

____

16.7 Do you have private medical insurance?

- ☐ No
- ☐ Yes

16.8 Have you experienced any of the following major life events in the last two years?

- ☐ Death of a spouse or partner
- ☐ Death of a close relative or friend
- ☐ Illness /accident of a family member
- ☐ Financial difficulties
- ☐ Personal illness, accident or injury
- ☐ Moving house
- ☐ Divorce
- ☐ Addition to family circle, for example, a grandchild
- ☐ Other ________ (please specify)
- ☐ None of these

16.9 Are you currently employed?

- ☐ No
- ☐ Yes

16.10 What kind of work do you do (or did you do in your most recent job) ?

Your main activity is/was: ________

16.11 Is your current or most recent job full-time or part-time?

- ☐ Full-time
- ☐ Part-time → 16.12 How many hours per week on average? [ ][ ]

16.13 In your current or most recent job: are you/were you self-employed?

- ☐ No
- ☐ Yes

16.14 If you are not currently employed, which of the following applies?

- ☐ Waiting to take up a job you've accepted
- ☐ Unemployed and seeking work
- ☐ Temporary sick/disabled
- ☐ Permanently sick/disabled
- ☐ House-wife/house-husband
- ☐ Not working for some other reason [ ] (please specify)
- ☐ Retired → At what age did you retire? [ ][ ] years of age

16.15 If you have a partner or spouse, what is his/her current or most recent job?

[ ]

16.16 At what age did you start school? [ ][ ] years of age

16.17 At what age did you finish your full-time education? [ ][ ] years of age

16.18 What is your highest level qualification? (please tick one only)

- ☐ No qualifications
- ☐ Don't know
- ☐ GCE 'O' levels/ GCSE/CSE or equivalent
- ☐ Apprenticeship
- ☐ ONC/OND/BTEC, NVQ level 3, City and Guilds advanced craft or equivalent
- ☐ HNC/HND, NVQ level 4-5, BTEC higher level or equivalent
- ☐ Professional qualification, for example teaching, nursing, accountancy
- ☐ Degree or higher degree, for example BA, BSc, MA, PhD)
- ☐ Other qualifications [ ] (please specify)

16.19 What is your household’s total gross income (before tax)?

Per week	or Per year (approximately)
Nil	☐ Nil
Up to £99	☐ Up to £5,199
£100 to £199	☐ £5,200 to £10,399
£200 to £299	☐ £10,400 to £15,599
£300 to £399	☐ £15,600 to £20,799
£400 to £499	☐ £20,800 to £25,999
£500 to £599	☐ £26,000 to £31,999
£600 to £999	☐ £31,200 to £51,999
£1000 or more	☐ £52,000 or more
	☐ I do not wish to answer this question

16.20 What level of financial stress or anxiety do you feel?

☐ Little / None

☐ Moderate

☐ High / Severe

Thank you very much for taking the time to fill in this questionnaire.

We very much appreciate your help

Please bring the questionnaire with you when you come to our clinic or return it to us in the reply paid envelope

SABRE Study-Freepost
UCL Institute of Cardiovascular Science
Gower Street
London WC1E 6BT

Tel: 020 7679 9471
